# A Nationwide Survey of Prevalence of Pediculosis in Children and Adolescents in Iran

**Published:** 2011-03-01

**Authors:** M A Amirkhani, S M Alavian, H Maesoumi, T Aminaie, M Dashti, G Ardalan, H Ziaoddini, P Mirmoghtadaee, P Poursafa, R Kelishadi

**Affiliations:** 1 Direcor General, Family, Health, Population and School Health Bureau, Ministry of Health and Medical Education, Baqiyatallah University of Medical Sciences, Tehran, Iran; 2 Department of Gastroentrology and Hepatology, Liver and Gastrointestinal Research Center, Baqiyatallah University of Medical Sciences, Tehran, Iran; 3 Department of Pediatric Infectious Diseases, Center for Disease Control, Ministry of Health and Medical Education, Tehran, Iran; 4 Senior Expert of Youth and School Health Office, Ministry of Health and Medical Education, Tehran, Iran; 5 Head of Adolescents, Youth and School Health Office, Ministry of Health and Medical Education, Tehran, Iran; 6 Department of Health and Physical Activity and Prevention of Social Hazards, Ministry of Health and Medical Education, Tehran, Iran; 7 Department of Community and Preventive Medicine, Isfahan University of Medical Sciences, Isfahan, Iran; 8 Environmental Protection Engineer, Islamic Azad University, Tehran Research and Science Branch, Tehran, Iran; 9 Department of Pediatrics, Pediatric Prevention Research Center, Isfahan University of Medical Sciences, Isfahan, Iran

**Keywords:** Pediculosis, Children, Prevalence, Iran

## Abstract

**Background:**

Since 2005, pediculosis is one of the obligatory reportable diseases from community to the Center of Disease Control. This study is the first nationwide survey on the prevalence of pediculosis and some associated risk factors in Iranian children and adolescents.

**Methods:**

National data of the Ministry of Health and Medical Education were gathered in 2005 through school screening programs and obligatory reports from the country health centers.

**Results:**

12,359,448 Iranian children and adolescents were screened in 2005. Overall, 213,450 students, consisting of 198,947 girls and 14,320 boys were reported to have pediculosis. The prevalence of pediculosis was 581 per 100,000 population that varied from 1/100 000 to 8,303/100,000. In general, the highest prevalence of pediculosis was documented in south-eastern cities. The prevalence of pediculosis was significantly higher in girls than in boys (93% vs.7%, respectively, p<0.0001). In both genders, the highest prevalence of pediculosis was documented in the 6-10- year age group. Of those infected, 62% lived in rural areas, and 32% of those infected with pediculosis had a previous history of this infection. Most (99.37%) infected individuals had head lice, the rest had body and pubic pediculosis.

**Conclusion:**

The prevalence of pediculosis is low in Iranian children and adolescents, but this infestation is still a health problem in some south-eastern cities with warm climate and low to middle socioeconomic status.

## Introduction

Pediculosis or louse infestation which is defined as an infestation with head, body or crab lice, remains a worldwide problem.[1][2][3] It mainly affects schoolchildren aged between 3 and 12 years.[1][2] Head lice infestations are the cause of much embarrassment and misunderstanding, many unnecessary days cost from school and work, and huge costs spent on remedies.[4] Given that its prevalence varies well according to the social situation, genetic and cultural characteristics of different populations, it is recommended to investigate it regionally.[5] Head lice infestation prevalence rates of 5.8% to 35% have been reported from different regions in different institutions.[6][7][8][9][10][11] In Iran, its prevalence was reported between 1.6% and 13.4% from some cities with various sociodemographic backgrounds.[12][13]

Since 2005, pediculosis is one of the obligatory reportable diseases from community to the Center of Disease Control and Iranian Ministry of Health and Medical Education (MoHME). It is also screened in all students attending first grade of elementary, middle and high schools, as well as in the 3rd grade of elementary schools.

In this study, we aimed to estimate the national prevalence of pediculosis and its differences by gender, living area and age group in Iranian children and adolescents.

## Materials and Methods

The national data of the MoHME were collected in 2005. Based on the national guidelines, from 2005, all health centers have to report pediculosis to the Center of Disease Control in each province, and in turn, the data is reported to the MoHME. The forms prepared by health officers are sent to all health care centers, to which all cases in the community are referred to. The screening method was by inspection. EPI info software, version 6 was used for statistical analysis.

## Results

A total number of 12,359,448 Iranian children and adolescents were screened in 2005 (81% of total population of students), which consisted of 5,648,811 elementary school students (first and third graders), 2,879,767 middle school students (first graders) and 3,830,870 high school students (first graders). Of the population studied, 213,450 students, consisting of 198,947 girls and 14,320 boys, were reported to have pediculosis. The prevalence of pediculosis was 581 per 100,000 population that varied from 1/100,000 (in Shiraz city) to 8,303/100,000 (in Kerman city). In general, the highest prevalence of pediculosis was documented in the south-eastern cities of Iran, such as Kerman, Bandar Abbas, Zahedan and Zabol.

The prevalence of pediculosis was significantly higher in girls than in boys (93% vs.7%, respectively, p<0.001). Overall, 2.2%, 61,7%, 29.8% and 6.3% of girls with pediculosis were aged under 6, 6-10, 11-17 and more than 17 years, respectively. In addition, 2.5%, 65.2%, 26.3% and 6% of boys with pediculosis were aged under 6, 6-10, 11-17 and more than 17 years, respectively (Table 1).

**Table 1:** Frequency of pediculosis in Iranian school students in 2005 by gender and age group.

**Age group (years)**	**Girls**		**Boys**		**Total**	
	**No.**	**%**	**No.**	**%**	**No.**	**%**
<6	4389	2.2	351	2.5	4740	2.2
6-10	122715	61.7	9338	65.2	132053	61.9
11-17	59372	29.8	3765	26.3	63137	29.6
>17	12471	6.3	866	6.0	13337	6.2
Total	198947	100	14320	100	213450	100

Of those affected with pediculosis, 38% lived in urban areas and 62% were rural resident. Overall, 9.2% of students with pediculosis studied in primary school, 3% in middle school and 3.2% in high school, and 32% of those infected with pediculosis had a previous history of this infection. Most (99.37%) infected individuals had head lice, the rest had body and pubic pediculosis.

## Discussion

This study is the first nationwide report on pediculosis not only from Iran, but to the best of our knowledge from the Eastern Mediterranean region. Most previous studies had estimated the prevalence of this infestation in specialized populations like day care centers and primary schools in small areas, and not at a community level. The prevalence of pediculosis encountered in the current study was 8,303/100,000 populations, and is among the lowest prevalence rates documented worldwide. The global prevalence of pediculosis is unknown; still it affects millions of population, especially children 5 to 14 years of age,[14] with the prevalence in primary schools averaging as high as 40% in some areas regardless of socioeconomic factors.[15] The low prevalence rate of pediculosis in our community might be because of nationwide strict screening programs in students and education of students and families about prevention and early detection of this infestation. In spite of the low prevalence documented, this communicable problem still exist in our community, and more intensive public health measures should be undertaken to control this infestation.

High prevalence of lice infestation in the southeastern parts of Iran might be due to the warm climate and low to middle socioeconomic level of inhabitants of these geographic regions. More intensive educational and hygienic programs should be implemented in these regions.

Our finding on the higher prevalence of pediculosis in girls than in boys is in line with many previous studies.[2][7][10][16] It is believed that gender-related behavior differences affect transmission rates, for instance difference in personal grooming close contact, hair style changes and the use of hair accessories.[17]

In our study, the proportion of infected children was different according to the age groups; these differences might be associated with behavioral variations in the different age groups.[5] However, many other investigators did not document any significant influence of age upon the incidence of lice infestation.[18][19]

The proportion of infected cases was higher in rural than in urban areas of Iran. This finding is consistent with some other studies,[20][21] and can be explained by the better cultural and hygienic indexes as well as higher educational level in urban than in rural inhabitants.

The main limitation of this study was that because of the very large number of individuals under study, we could not have access to some variables like socioeconomic variables, family size, and hair style and length that might be associated with pediculosis rate. The strength of this study is its population-based methodology and covering all children and adolescents in Iran in 2005 at a national level.

Our findings showed that the prevalence of pediculosis was low in Iranian children and adolescents, but this infestation is still a health problem in some south-eastern cities with warm climate and low to middle socioeconomic situation. The school health system plays a pivotal role for students and families by organizing information campaigns and practice sessions about the prevention, screening and treatment of pediculosis in a timely manner.
